# MicroRNA-200b and microRNA-200c are up-regulated in PCOS granulosa cell and inhibit KGN cell proliferation via targeting PTEN

**DOI:** 10.1186/s12958-019-0505-8

**Published:** 2019-08-17

**Authors:** Tingting He, Yifei Sun, Yingchun Zhang, Shigang Zhao, Yanjun Zheng, Guimin Hao, Yuhua Shi

**Affiliations:** 1 Center for Reproductive Medicine, Shandong University; The Key Laboratory of Reproductive Endocrinology (Shandong University), Ministry of Education, China; Shandong Provincial Clinical Medicine Research Center for reproductive health, Jinan, 250021 China; 2 Reproductive Medicine Center, The Northwest Women’s & Children’s Hospital affiliated of Xi’an Jiaotong University, Xi’an, 710003 China; 3 Department of Reproductive Medicine, the Second Hospital of Hebei Medical University, Shijiazhuang, Hebei China; 4 Department for Reproductive Medicine, Jinan Central Hospital, Affiliated to Shandong University, Jinan, Shandong China

**Keywords:** Polycystic ovary syndrome, microRNA-200b, microRNA-200c, Proliferation, PTEN

## Abstract

**Background:**

Polycystic ovary syndrome (PCOS) is one of the most common endocrine metabolic disorders characterized by hyperandrogenism, polycystic ovaries and ovulatory dysfunction. Several studies have reported that the aberrant expression of miRNAs contributes a lot to disordered folliculogenesis in PCOS, though the role and underlying mechanism of microRNA-200b (miR-200b) and microRNA-200c (miR-200c) in the development of PCOS remain unclear.

**Methods:**

The expression of miR-200b in granulosa cells (GCs) derived from 90 PCOS patients and 70 controls was analyzed by using quantitative reverse transcription-polymerase chain reaction (qRT-PCR). Granulosa-like tumor cell line (KGN) was cultured for cell counting kit-8 (CCK-8) assays after over-expression of miR-200b, miR-200c or knockdown phosphatase and tensin homolog (PTEN). TargetScan was used to identify the potential targets of miR-200b and miR-200c, which was further verified by qRT-PCR, western blot and luciferase assays.

**Results:**

Significantly increased expression of miR-200b was observed in PCOS patients compared with the controls. Moreover, over-expression of miR-200b and miR-200c inhibited the proliferation of KGN cells. In addition, our results verified that miR-200b and miR-200c directly targeted PTEN, knockdown of which suppressed KGN cells proliferation.

**Conclusion:**

Our findings demonstrate that miR-200b and miR-200c suppress the proliferation of KGN cells by targeting PTEN, and this might provide new evidence for abnormal proliferation of GCs in PCOS.

## Background

Polycystic ovary syndrome (PCOS) is one of the most common endocrine metabolic disorders affecting about 5% women of reproductive age [1]. It is characterized by hyperandrogenism, polycystic ovaries and ovulatory dysfunction, but also associated with insulin resistance, cardiovascular risk, obesity, abnormal granulosa cells (GCs) proliferation as well as arrest of follicle growth [2–4]. It has been estimated that genetic and environmental factors play an important role in the pathogenesis of PCOS, but the underlying molecular mechanism is still unclear [5, 6].

A distinctive feature of PCOS is the accumulation of preantral and antral follicles exceeding by 2–3 fold that of normal ovaries, and this might be due to the arrest of follicle growth typically occurs when follicular diameter reaches 5–8 mm [7–9]. Throughout oocyte development, it has been demonstrated that there is an interdependence between the oocyte and its surrounding GCs, the support of which is essential to provide the oocyte with a suitable microenvironment, such as nutrients and growth regulators [10–12]. Moreover, recent reports confirm that the dysfunction of GCs in PCOS ranging from decreased proliferation and increased apoptosis to hormone production disorders is closely associated with abnormal folliculogenesis [13].

MicroRNAs (miRNAs) are a class of single-stranded and non-coding RNAs (20–24 nucleotides), which down-regulate the expression of target genes in a post-transcriptional manner by binding to the 3′-untranslated region (UTR) of target mRNA [14]. Studies have shown that aberrant expressions of miRNAs are associated with the pathological progression of various diseases, including cancer, metabolic diseases and reproductive disorders [15–17]. Furthermore, miRNAs are revealed to be involved in the regulation of proliferation, apoptosis and steroidogenesis in GCs, the dysregulation of which may play an important role in the pathogenesis of PCOS [18, 19]. Our recent study has demonstrated that miR-200c was dramatically increased in GCs of PCOS [20]. However, there is little known about the functional role of miR-200b and miR-200c in the development of PCOS. Therefore, the purpose of this study is to explore whether miR-200b and miR-200c are involved in the abnormal proliferation of PCOS GCs and its underlying mechanism.

## Methods

### Clinical samples

A total of 160 participants (90 PCOS and 70 controls) who underwent intracytoplasmic sperm injection (ICSI) or in vitro fertilization (IVF) at the Center for Reproductive Medicine, Shandong University were included. This research was approved by the Institutional Review Board of Center for Reproductive Medicine of Shandong University and formal written consent was obtained from each patient. PCOS was diagnosed according to the revised Rotterdam consensus, and women with regular menstruation and normal ovarian function served as controls [21]. The clinical and endocrine parameters of PCOS patients and controls were analyzed and presented in Table 1. GCs were collected from each participant as described previously, and then immediately stored at − 80 °C for further analysis [22].

**Table 1:** Clinical and endocrine parameters of PCOS patients and controls

Basic parameters	PCOS (*n* = 90)	Control (*n* = 70)	*P* value
Age (years)	28.30 ± 3.01	28.65 ± 2.42	NS
BMI (kg/m^2^)	24.40 ± 3.62	21.75 ± 2.45	< 0.001
FPG (mmol/L)	5.41 ± 0.44	5.17 ± 0.43	< 0.001
FINS (mIU/L)	15.31 ± 7.79	7.87 ± 1.94	< 0.001
LH (IU/L)	8.29 ± 3.80	4.95 ± 1.43	< 0.001
FSH (U/L)	5.88 ± 1.05	6.48 ± 1.09	< 0.001
T (ng/dL)	39.02 ± 15.69	22.63 ± 7.48	< 0.001
AMH (ng/ml)	9.28 ± 4.22	4.05 ± 1.83	< 0.001
AFC (mmol/l)	26.10 ± 8.87	13.02 ± 3.52	< 0.001

Data were presented as mean ± SD

### Cell culture

Asteroidogenic human granulosa-like tumor cell line, KGN (a gift from RIKEN BioResource Center, Ibaraki, Japan), maintained the physiological characteristics of ovarian cells [23]. The cells were grown in DMEM/F12 (HyClone) supplemented with 10% FBS (HyClone) and 1% antibiotics (HyClone), while the human embryonic kidney (HEK) 293 T cell line was cultured in DMEM High Glucose (HyClone) supplemented with 10% FBS and 1% antibiotics. All cells were cultured in a humidified atmosphere containing 5% CO_2_ at 37 °C.

### Cell transfection

MiR-200b mimics, miR-200b inhibitor, miR-200c mimics, miR-200c inhibitor, mimics control, inhibitor control and specific small-interfering RNA (siRNA) for phosphatase and tensin homolog (PTEN) were designed and synthesized by Boshang (jinan, China). The transfection of miRNAs and siRNA was performed with X-tremeGENE siRNA Transfection Reagent (Roche) according to the manufacturer’s instructions at 100 nM and 50 nM respectively. The transfected cells were incubated at 37 °C and harvested at the indicated time points (24 h or 48 h) for the following assays.

### RNA extraction and qRT-PCR

In order to verify the expression of PTEN at mRNA level, total RNA was extracted from cells by using TRIzol Reagent (Invitrogen) and reversely transcribed into cDNA with PrimeScript RT reagent Kit With gDNA Eraser (Takara) according to the manufacturer’s instructions. However, the RNA extracted by miRNeasy Mini Kit (Qiagen) was reversely transcribed into cDNA using MiRNA-X miRNA First-Strand Synthesis Kit (TaKaRa) for microRNA verification. Then, qRT-PCR was performed on a Light Cycler 480 system by using SYBR Premix Ex Taq (Takara) according to the manufacturer’s instructions. U6 and ACTIN were used to normalize the expression of miRNAs and PTEN respectively. The relative expression was calculated using the 2^−△△CT^ method and the primers were listed in Table 2.

**Table 2:** Primer sequences for qRT-PCR

	Primer Sequences
microRNA-200b-3p	5’GCTAATACTGCCTGGTAATGATGA3’
microRNA-200c-3p	5’CTAATACTGCCGGGTAATGATGGA3’
U6	F: 5’GCTTCGGCAGCACATATACTAAAAT3’
	R: 5’CGCTTCACGAATTTGCGTGTCAT3’
PTEN	F: 5’TGGATTCGACTTAGACTTGACCT3’
	R: 5’GGTGGGTTATGGTCTTCAAAAGG3’
ACTIN	F: 5’TTCGAGCAAGAGATGGCCA3’
	R: 5’CGTACAGGTCTTTGCGGAT3’

### Western blot

After treatment, total protein was harvested in 1 × SDS loading buffer and equal amounts of protein were separated by sodium dodecyl sulfate polyacrylamide gel (SDS-PAGE). The polyvinylidene fluoride (PVDF) membranes (Millipore, USA) transferred with bands were blocked with 5% milk and then incubated with primary antibodies at 4 °C overnight. After the membranes were incubated with peroxidase-conjugated secondary antibodies (Zhongshan, Beijing, China) for 1 h at room temperature, BIO-RAD ChemiDoc MP Imaging System and Image Lab Sofware were used to detect and analyze immunoreactive bands. The primary antibodies for immunoblotting included anti-PTEN (Proteintech, 60300–1-Ig) and anti-ACTIN (Cell Signaling Technology, 4970 s).

### Cell counting kit-8 (CCK-8)

KGN cells transfected with miRNAs or siRNA for 24 h were reseeded in 96-well plates at 4000 cells/well. Then, cell proliferation ability was assessed using the CCK-8 assay (Beyotime, China) according to the manufacturer’s instructions at 0, 24 and 48 h respectively.

### Luciferase reporter assay

Wild type (WT) and mutant type (MUT) recombinant reporter plasmids of PTEN were synthesized by GeneCopoeia, Guangzhou, China. These plasmids were co-transfected with miR-200b mimics, miR-200c mimics or mimics control into HEK293T cells using X-tremeGENE siRNA Transfection Reagent. After transfection for 48 h, cultured supernatant was collected and measured by Secrete-Pair™ Dual Luminescence Assay Kit (Genecopoeia) according to the manufacturer’s instructions.

### Statistical analysis

All statistical analyses were performed using SPSS 21.0 (SPSS, Chicago, IL, USA), and data were presented as mean ± standard deviation (SD). Kolmogorov–Smirnov was used to assess whether the data were of normal distribution. Normally distributed variables were analyzed by Student’s *t*-test to determine statistical significance, while nonparametric data were assessed using the Mann-Whitney U test. Logistic regression was used to adjust age and BMI to avoid their potential effects on the expression of miR-200b. *P* < 0.05 was considered statistically significant (*P < 0.05; **P < 0.01; ***P < 0.001).

## Results

### Clinical and endocrine parameters of PCOS patients and controls

The clinical and endocrine parameters of PCOS patients and controls were listed in Table 1. Compared to controls, BMI, FPG, FINS, LH, T, AMH and AFC were significantly increased in PCOS patients, while FSH was dramatically decreased (*P* < 0.05, for all). There was no difference in term of age between the two groups (*P* > 0.05).

### The expression of miR-200b in PCOS GCs

**Fig. 1:**
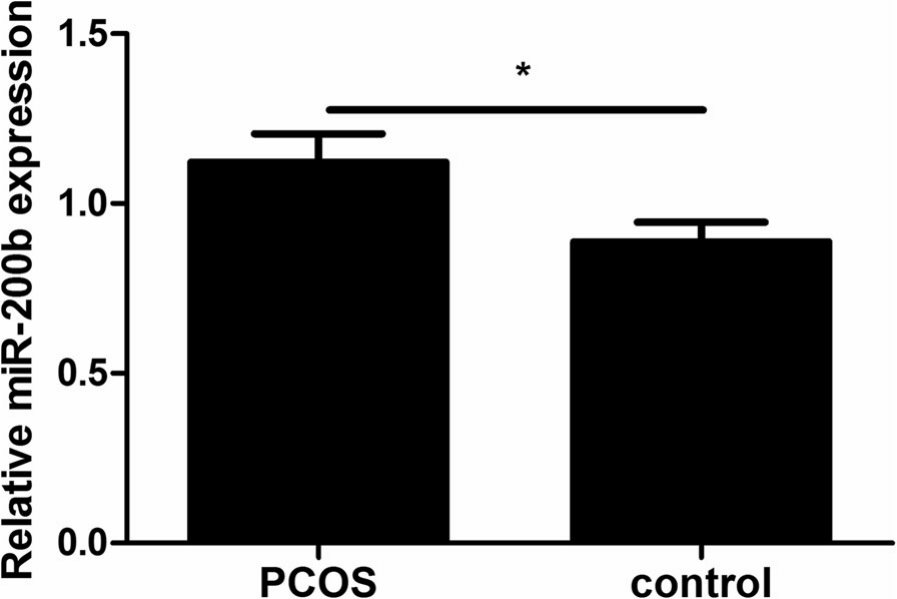
The expression of miR-200b was significantly increased in GCs of PCOS patients. The expression level was detected by qRT-PCR and normalized against U6. Statistical analysis was performed by Student’s *t*-test. Logistic regression was used to adjust age and BMI to avoid their potential effects on the expression of miR-200b. Data were shown as the mean ± SD. *P < 0.05

We have previously demonstrated that the level of miR-200c was increased in PCOS GCs [20]. To determine whether miR-200b was also associated with PCOS, qRT-PCR was used to assess the expression of miR-200b in GCs derived from 90 PCOS and 70 control women. The results showed that the level of miR-200b was also significantly increased in PCOS after adjustment age and BMI (*P* < 0.05; Fig. 1).

### MiR-200b and miR-200c suppressed cells proliferation

**Fig. 2:**
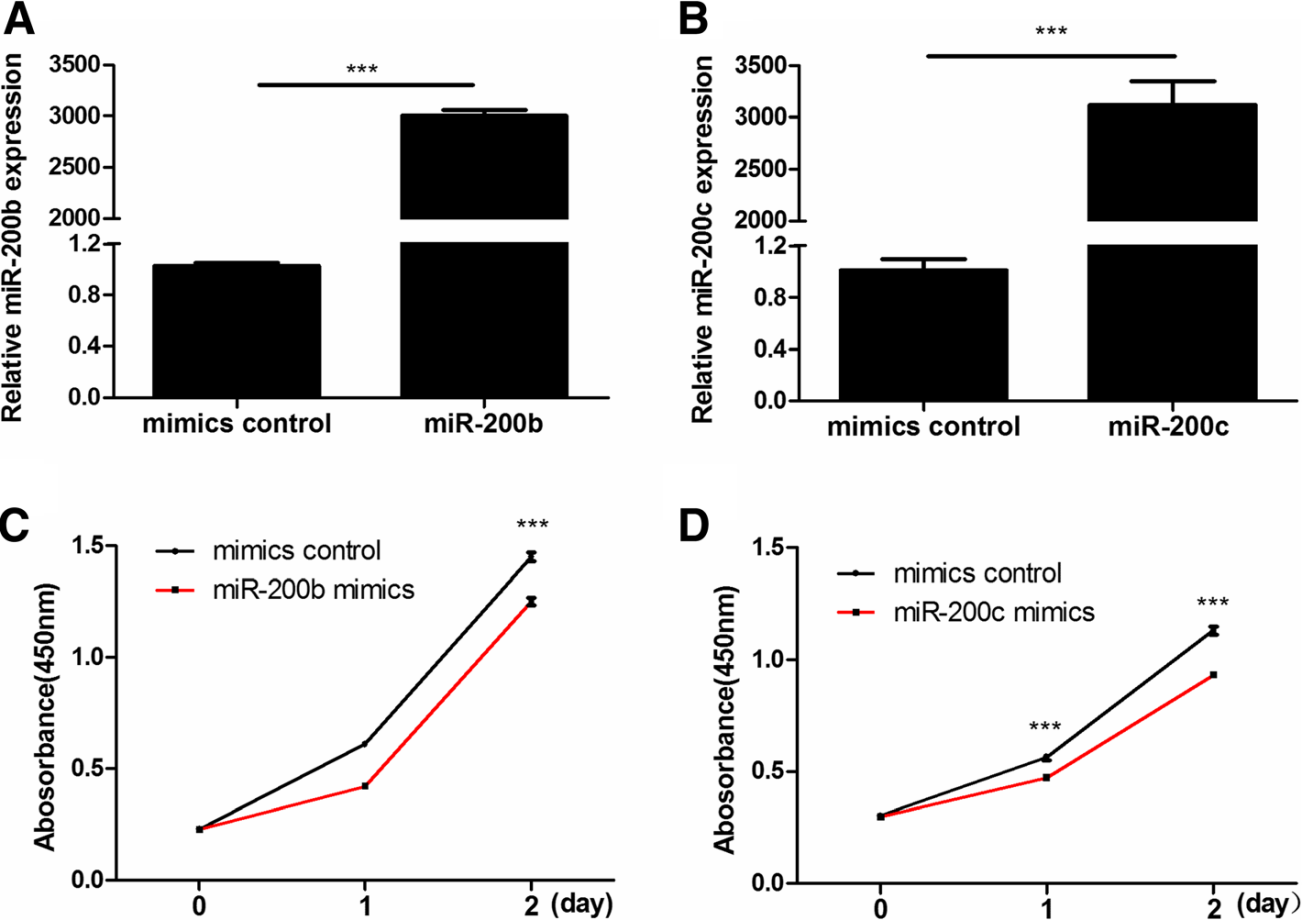
Over-expression of miR-200b and miR-200c inhibited KGN cells proliferation. (**a**) and (**b**) The expression levels of miR-200b and miR-200c in KGN cells after transfection with miR-200b or miR-200c mimics. The expression level was detected by qRT-PCR and normalized against U6 (*n* = 3). (**c**) and (**d**) The proliferation ability of KGN cells transfected with miR-200b or miR-200c mimics was determined by CCK-8 assays (*n* = 6). The results were representative of three independent experiments. Statistical analysis was performed by Student’s *t*-test. Data were shown as the mean ± SD. ***P < 0.001

To verify the hypothesis that miR-200b and miR-200c might be involved in GCs proliferation, KGN cells were transiently transfected with miR-200b mimics, miR-200c mimics or mimics control. The validation of miR-200b and miR-200c over-expression through mimics was showed in Fig. 2a-b. As illustrated in Fig. 2c-d, CCK-8 assays revealed that up-regulation of miR-200b or miR-200c resulted in an inhibition of KGN cells proliferation (*P* < 0.05, for all).

### PTEN was a direct target of miR-200b and miR-200c

**Fig. 3:**
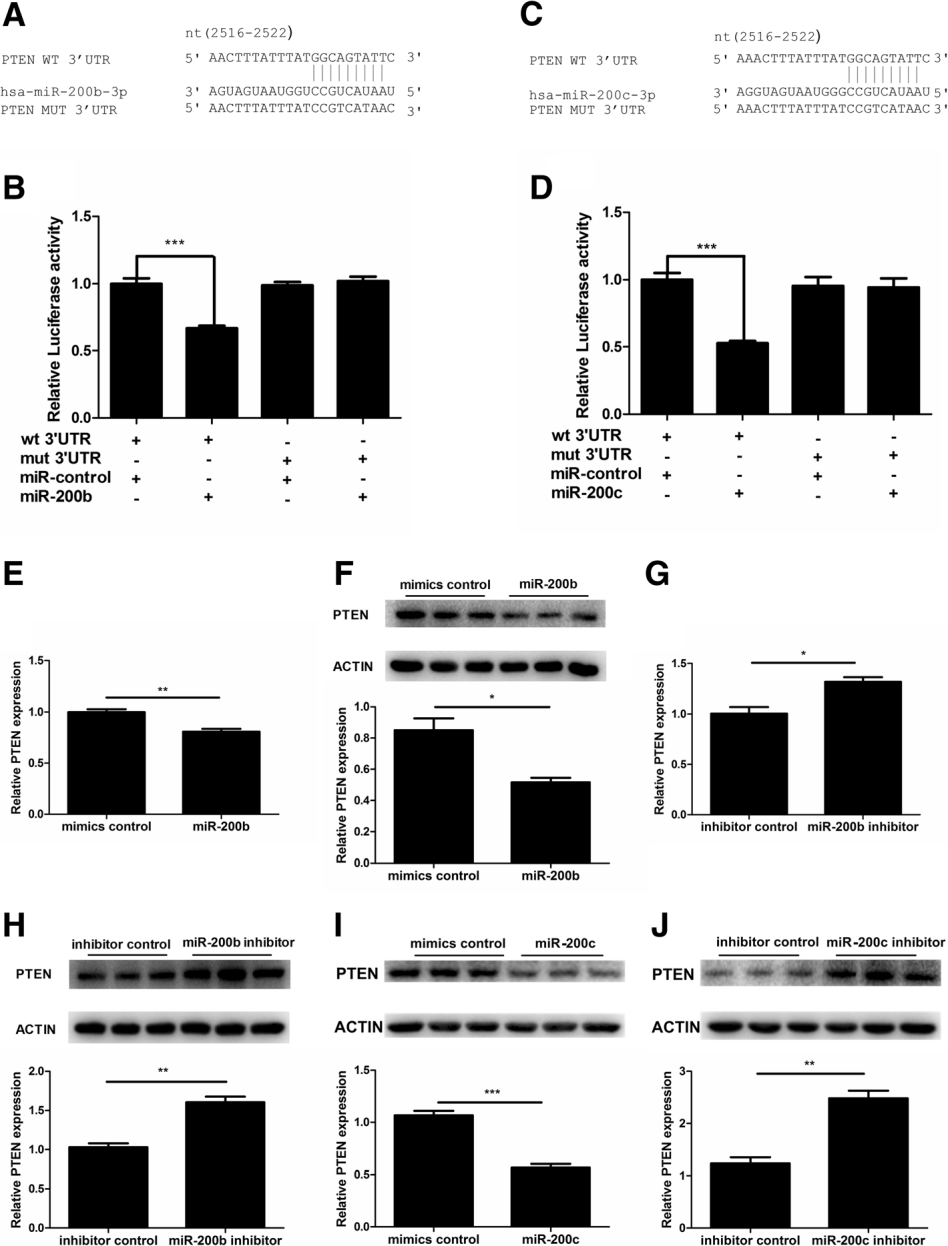
PTEN was a direct target of miR-200b and miR-200c in KGN cells. (**a**) and (**c**) Schematic diagram of the reporter constructs containing the predicted miR-200b and miR-200c binding sites in the 3’UTR of PTEN. (**b**) and (**d**) The luciferase activity was significantly decreased in HEK293T cells by co-transfecting miR-200b or miR-200c mimics with PTEN WT 3’UTR, while there was no change with PTEN MUT 3’UTR (*n* = 6). (**e**) and (**f**) The mRNA and protein levels of PTEN after miR-200b mimics transfection (*n* = 3). (**g**) and (**h**) The mRNA and protein levels of PTEN after miR-200b inhibitor transfection (*n* = 3). (**i**) and (**j**) The protein levels of PTEN after miR-200c mimics or inhibitor transfection (*n* = 3). The results were representative of three independent experiments. Statistical analysis was performed by Student’s *t*-test. Data were shown as the mean ± SD. *P < 0.05; **P < 0.001; ***P < 0.001

To investigate the mechanism underlying the proliferation-suppressive role of miR-200b and miR-200c in GCs, we searched for their potential target genes using TargetScan. PTEN, which was closely related to cell proliferation, provoked our interest. In order to verify the prediction of TargetScan, we constructed luciferase reporter vectors containing the predicted binding site of PTEN 3’UTR. The results demonstrated that over-expression of miR-200b or miR-200c led luciferase activity significantly decreased by co-transfecting with PTEN WT 3’UTR, while there was no change with PTEN MUT 3’UTR (*P* < 0.05, *P* > 0.05; Fig. 3a-d). To further investigate the regulation of miR-200b and miR-200c on PTEN, qRT-PCR and western blot were performed. Our results demonstrated that over-expression of miR-200b via miR-200b mimics decreased the mRNA and protein expressions of PTEN (*P* < 0.05, for all; Fig. 3e-f), whereas miR-200b inhibitor led PTEN mRNA and protein levels up-regulated compared with inhibitor control (*P* < 0.05, for all; Fig. 3g-h). In addition, miR-200c mimics significantly inhibited PTEN protein level, while miR-200c inhibitor remarkably increased PTEN protein level (*P* < 0.05, for all; Fig. 3i-j). Taken together, these results suggest that PTEN is a direct target of miR-200b and miR-200c in KGN cells.

### Knockdown of PTEN inhibited cell proliferation

**Fig. 4:**
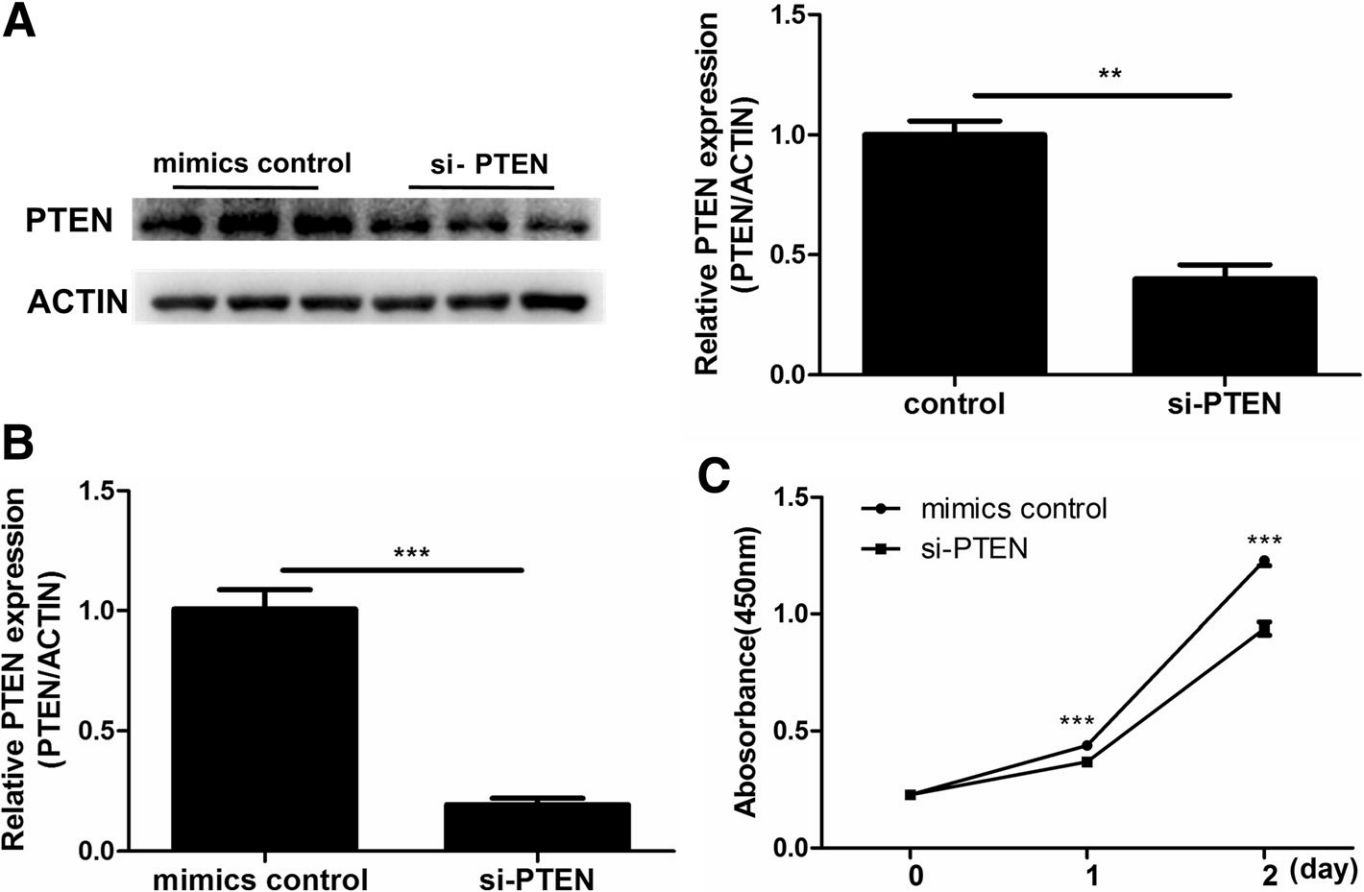
Down-regulation of PTEN suppressed KGN cells proliferation. (**a**) and (**b**) The mRNA and protein levels of PTEN in KGN cells after si-PTEN transfection (*n* = 3). (**c**) The CCK-8 assay was performed to measure KGN cells proliferation after si-PTEN transfection (*n* = 6). The results were representative of three independent experiments. Statistical analysis was performed by Student’s *t*-test. Data were shown as the mean ± SD.***P < 0.001

To determine whether PTEN had an effect on cell proliferation, we knocked down it in KGN cells via specific siRNA. Western blot and qRT-PCR confirmed that si-PTEN significantly decreased PTEN mRNA and protein levels in KGN cells after 48 h post-transfection (*P* < 0.05, for all; Fig. 4a-b). Thereafter, a CCK-8 assay was performed at 24 h and 48 h respectively, and it was found that down-regulation of PTEN significantly inhibited the proliferation of KGN cells, which resembled the suppressive effect of miR-200b or miR-200c over-expression (*P* < 0.05, for all; Fig. 4c).

## Discussion

The aim of this study was to explore whether miR-200b and miR-200c were involved in the abnormal proliferation of PCOS GCs and its underlying mechanism. And our results demonstrated that the expression of miR-200b was significantly increased in PCOS patients, and over-expression of miR-200b and miR-200c inhibited the proliferation of KGN cells. In addition, PTEN was a direct target of miR-200b and miR-200c in KGN cells, knockdown of which revealed similar proliferation-inhibiting effect as observed when miR-200b and miR-200c were over-expression.

Increased expression of miR-200b was observed in PCOS GCs, and the previous study showed that the expression of miR-200c was also up-regulated, both of which contributed a lot to insulin resistance, one of the typical characteristics of PCOS [20, 24, 25]. In addition, miR-200b and miR-200c were closely related to proliferation. Li Y et al. reported that miR-200b inhibited the proliferation of osteosarcoma cells via targeting ZEB1, while another study indicated that over-expression of miR-200c significantly suppressed cells proliferation in lung cancer [26, 27]. However, a large number of studies about miR-200b and miR-200c were mainly focused on cancer, while it was relatively limited in PCOS. This was the first time to confirm that miR-200b and miR-200c were involved in inhibiting the proliferation of KGN cells, suggesting that both miR-200b and miR-200c play crucial roles in the abnormal proliferation of GCs, which might lead to PCOS.

It has been reported that PTEN was a direct target of miR-200b and miR-200c in endometrial cancer and nasopharyngeal carcinoma [28, 29]. In addition, a growing number of studies indicated that PTEN played an important role in the abnormal proliferation of PCOS GCs [30, 31]. In consistent with the above results, our research further proved that PTEN was a direct target of miR-200b and miR-200c in KGN cells, and down-regulation of PTEN suppressed KGN cells proliferation. These findings, along with our previous observation of dramatically decreased expression of PTEN in PCOS GCs, suggest that PTEN might be responsible for the decreased proliferation potential of GCs in PCOS, and leads them prone to apoptosis, followed by follicular atresia [32, 33]. However, Andreas E et al., who investigated the role of miR-17-92 cluster in bovine GCs, demonstrated that down-regulation of PTEN promoted the proliferation of GCs [34]. The differences were likely due to different sources of cells. The GCs studied above were isolated from bovine small follicles (3–5 mm), while KGN cells used in our study were a steroidogenic human granulosa-like tumor cell line maintaining similar physiological characteristics to that of human immature GCs [23]. In addition, as far as we know, the regulation process of GCs proliferation was complex and closely related to various factors [35].

This is a pioneering study to elucidate the functions of miR-200b, miR-200c and PTEN in PCOS GCs and their relationships. In the meantime, there are two potential limitations in our study. On the one hand, a large number of PCOS patients are required to further verify the expressions of miR-200b, miR-200c and PTEN. On the other hand, it would be more conclusive and convincing if PTEN reintroduction could reverse the proliferation-suppressive roles of miR-200b and miR-200c. Therefore, we plan to continue this experiment in the following work.

## Conclusions

In conclusion, we found that the expression of miR-200b was significantly increased in PCOS patients. In addition, over-expression of miR-200b and miR-200c inhibited the proliferation of KGN cells by targeting PTEN. These results provide new evidence for GCs dysregulation proliferation observed in PCOS. However, further studies are needed to investigate the underlying molecular mechanisms.

## Data Availability

Data sharing is not applicable to this article as no datasets were generated or analysed during the current study.

## Abbreviations

AFC: Antral follicle count
AMH: Anti-Mullerian hormone
BMI: Body mass index
CCK-8: Cell counting kit-8
FINS: Fasting insulin
FPG: Fasting plasma glucose
FSH: Follicle stimulating hormone
GCs: Granulosa cells
ICSI: Intracytoplasmic sperm injection
IVF: In vitro fertilization
LH: Luteotropic hormone
miR-200b: microRNA-200b
miR-200c: microRNA-200c
miRNAs: microRNAs
MUT: Mutant type
NS: Not significant
PCOS: Polycystic ovary syndrome
PTEN: Phosphatase and tensin homolog
qRT-PCR: Quantitative reverse transcription-polymerase chain reaction
SD: Standard deviation
siRNAs: specific small-interfering RNAs
T: Testosterone
UTR: Untranslated region
WT: Wild type
